# Effect of liquid volume and microflora source on degradation rate and microbial community in corn stover degradation

**DOI:** 10.1186/s13568-021-01233-5

**Published:** 2021-06-01

**Authors:** Jingjing Wang, Dan Zhu, Siqi Zhao, Song Xu, Rong Yang, Wei Zhao, Xiaoxia Zhang, Zhiyong Huang

**Affiliations:** 1 Tianjin Key Laboratory for Industrial Biological Systems and Bioprocessing Engineering, Tianjin Institute of Industrial Biotechnology, Chinese Academy of Sciences, No. 32, West 7th Avenue, Tianjin Airport Economic Area, Tianjin, 300308 P R China; 2 National Technology Innovation Center of Synthetic Biology, Tianjin, 300308 China

**Keywords:** Corn stover degradation, Microbial consortia, Microbial diversity, Dissolved oxygen, Cellulase

## Abstract

Degradation is the bottleneck in the utilization of crop straw. In this paper, we screened the microbial consortia degrading corn stover from straw degrading consortia MC1 (M), sheep feces (Y), and mixtures (Q) of M, Y, and cattle feces. The effects of microflora source and liquid volume (representing dissolved oxygen) on the microbial community and degradation rate of corn stover were investigated. The results showed that the degradation rate and cellulase activity of a 200 mL liquid volume (L2) were significantly higher than that of 100 mL (L1). Microflora source had a significant effect on bacterial and fungal diversity, composition and taxa. Q and Y had higher bacterial and fungal α-diversity than that of M. The degradation rate was significantly correlated with cellulase activity but not with microbial diversity. This indicated that liquid volume had a significant effect on degradation rate while microflora source had a significant effect on microbial community in corn stover degradation.

## Key points

- Liquid volume had a significant effect on degradation rate and cellulase activity.
- Microflora source had a significant effect on microbial community.
- The degradation rate was significantly correlated with cellulase activity.

## Introduction

Crop straw is the most abundant renewable biomass, and its worldwide annual yield is estimated at 200 billion tons (Liang et al. 2020). The use of crop straw as a source of energy, forage, fertilizer, and other high value chemicals is of great interest (Zhang 2008). However, due to the complexity of lignocellulose structure, degradation is the bottleneck in the utilization of crop straw. Compared with the physical and chemical degradation of straw, microbial degradation has the advantages of environmental friendliness and high efficiency (Liang et al. 2020). A number of microorganisms have been isolated and used to degrade straw (Arntzen et al. 2020; Ding et al. 2019). Under natural conditions, the degradation of straw depends on the synergistic action of multiple microorganisms (Liang et al. 2020; Wang et al. 2016). Microbial consortia can improve the efficiency and stability of straw degradation compared to a single strain (Gong et al. 2020; Zuroff and Curtis 2012). Many microbial consortia with high cellulose-degrading activity have been obtained by combination or domestication (Chu et al. 2021; Kato et al. 2005).

Corn stover accounts for about 25 % of crop straw. There are many microbial consortia that can degrade corn stover (Table 1). Some microbial consortia can degrade more than 70 % of corn stover pretreated with acid or alkali (Wongwilaiwalin et al. 2010; Zhang et al. 2018). However, this is not only expensive but also pollutes the environment. Most microbial consortia can only degrade less than 60 % of corn stover without pretreatment (Yu et al. 2019; Zhang et al. 2012). Therefore, it is necessary to screen the efficient microbial consortia, which can degrade corn stover without pretreatment.

**Table 1:** Microbial consortia degrading corn stover

Microbial Consortia	Temperature (°C)	Speed (rpm)	Pretreated	Degradation ratio (%)	Time (days)	Reference
CDS-10, enriched from rotten animal manure and corn straw	25	180	1.5 % H_2_SO_4_	63.09	15	(Tang et al. 2020)
Consisting of *Pelomonas* gx. and *Curvibacter* zj.	35	120	10 % NaOH	78.10	15	(Zhang et al. 2018)
Enriched from corn field soil	30	0	1.5 % NaOH	66.1	10	(Deng et al. 2017)
BGC-1, enriched from industrial sugarcane bagasse pile	50	200	10 % NaOH	72	4	(Wongwilaiwalin et al. 2013)
Enriched from feces and sludge	50	0	Steam-exploded	62	7	(Zhang et al. 2012)
CSS-1, enriched from sugarcane bagasse compost	50	0	Alkali-peracetic acid	70	7	(Wongwilaiwalin et al. 2010)
Consisting of three *Streptomyces*	30	210	NO	60.55	7	(Gong et al. 2020)
GF-20, enriched from soil and cow dung	30	0	NO	59.47	60	(Qinggeer et al. 2016; Yu et al. 2019)
Consisting of *Pelomonas* gx. and *Curvibacter* zj.	35	120	NO	58	15	(Zhang et al. 2018)
Enriched from the soil of a cattle and chicken manure storage tank	28–32	80	NO	48.52	6	(Wang et al. 2014)
Enriched from feces and sludge	50	0	NO	51	7	(Zhang et al. 2012)
Enriched from straw accumulation soil and rotten straw	37	0	NO	40	50	(Qiao et al. 2013)
MC1, enriched from compost	55	0	NO	59	14	(Cui et al. 2002; Yuan et al. 2011)
H-C, enriched from woodlands soil	40	0	NO	51	8	(Feng et al. 2011)
CSS-1, enriched from sugarcane bagasse compost	50	0	NO	62	7	(Wongwilaiwalin et al. 2010)
CSS-1, enriched from corn field soil	30	0	NO	40.9	16	(Liu et al. 2010)

Efficient straw degrading microflora are usually obtained from ruminant feces or long-term storage of lignocellulose (Haruta et al. 2002; Liang et al. 2020). Wongwilaiwalin et al. (2013) demonstrated that the microflora sources had significant effects on degradation rate and microbial community. Xing et al. (2020) demonstrated that cow rumen microorganisms are more suitable than sheep rumen microorganisms for corn stover transformation. Efficient straw-degrading microbial consortia mostly depend on the efficient cooperation of aerobic and anaerobic bacteria (Kato et al. 2005; Zhou et al. 2015). Generally, aerobic bacteria consume oxygen and provide a suitable living environment for anaerobic bacteria. Anaerobic bacteria provide a carbon source for aerobic bacteria, mainly by degrading lignocellulose. Some literature has shown that oxygen significantly affects the efficiency of straw degradation (Lu et al. 2008; Wang et al. 2004). However, the effect of oxygen on the microbial community for corn stover degradation has not been reported.

In this paper, we used the domestication method to screen the microbial consortia that can efficiently degrade corn stover without pretreatment from different environments and studied the effects of microflora source and dissolved oxygen (reflected by liquid volume) on the degradation rate and microbial community of corn stover. This can not only provide guidance for screening efficient straw degradation community but also lay a foundation for mechanism analysis of microbial community degradation of corn stover.

## Materials and methods

### Materials

Sources of microflora for the preparation of microbial consortia in this study were collected from (1) MC1, which was domesticated to degrade rice straw (Haruta et al. 2002), (2) sheep feces from a sheep farm (Shijiazhuang, China), and (3) cattle feces from a cow farm (Shijiazhuang, China). Chopped corn stover (the length was about 2–10 cm, and the width was about 0.2-2 cm) was obtained from Jilin, China.

### Construction of microbial consortia degrading corn stover

Ten grams (or 10 mL) of MC-1, sheep feces, or cattle feces was used to inoculate a 250 mL flask containing unsterilized 100 mL PCS media (0.1 % yeast extract, 0.5 % peptone, 0.5 % CaCO_3_, 0.5 % NaCl, and 2 % corn stover) (Haruta et al. 2002). The mixture was incubated at 50 °C under static conditions for 20 days, after which 10 mL of the culture was then transferred into fresh media. This procedure was repeated 3 times. After that, we obtained microbial consortia degrading corn stover from MC1 (M), sheep feces (Y), and cattle feces (N). Then, the three consortia were equally mixed together to obtain microbial consortia Q.

### Successive subcultivation of microbial consortia degrading corn stover

Efficient microbial consortia M (M), microbial consortia Q (Q), microbial consortia Y (Y), and PCS medium (CK) were used to inoculate a 250 mL conical flask containing 4 g corn straw and 100 mL sterilized PCS media at 20 % inoculum (L1), and then incubated for 25 days at 50 °C for 3 consecutive generations. The 20 % inoculum was used to inoculate a 250 mL conical flask containing 4 g corn stover and 200 mL sterilized PCS media (L2), and incubated at 50 °C for 25 days. The initial surface dissolved oxygen was detected by Luminescent Dissolved Oxygen (LDO) Sensors HQ40d (HACH Company, Loveland, Colorado, USA).

### Determination of the degradation rate of corn stover

The 9 samples cultured from three microbial consortia (M/Q/Y) three times (3–5 generations) were shaken and filtered aseptically. The filtrate was mixed with 50 % glycerin (1:1) and stored at − 80 °C for analysis. The straw residue was washed twice with 3 % acetic acid and water, and then dried in an oven at 105 °C (Li et al. 2020). The degradation rate was calculated by dividing the residual weight in treatments by that in CK.

### Analysis of cellulase activity

Endo-glucanase (CMCase, Endo-1, 4-b-d-glucanase; EC 3.2.1.4) activity of the 9 samples was analyzed by following the method of Saini et al. (2015). Briefly, 0.5 mL of suitably diluted filtrates and 1 mL of 1 % (w/v) CMC solution in citrate buffer (50 mM, pH 4.5) were mixed and incubated at 50 °C for 30 min. The reaction was terminated by adding 1 mL of 1 mol/L NaOH solution. Then, 3 mL of 3,5-dinitrosalicylic acid (DNS) was added and incubated in boiling water for 10 min. After cooling with running water, the volume was fixed to 25 mL, and the absorbance of glucose was measured at 540 nm. One unit (IU) of enzyme activity was defined as the amount of enzyme required to liberate 1 µmol of glucose.

### Analysis of microbial diversity

The filtrates of the 9 samples were used to extract DNA and sequence using bacterial (515 F: GTGCCAGCMGCCGCGGTAA; 806R: GGACTACHVGGGTWTCTAAT) and fungal (ITS5-1737 F: GGAAGTAAAAGTCGTAACAAGG; ITS2-2043R: GCTGCGTTCTTCATCGATGC) primers using the Hiseq platform by Novogen Co., Ltd (Tianjin, China). Microbial diversity analysis was performed using BMKCloud (www.biocloud.net). All statistical analyses were performed using R (version 3.1.1). Analysis of variance (ANOVA) was used to evaluate the effects of microflora source and liquid volume on corn stover degradation, cellulase activity, and microbial diversity. Principal coordinate analysis (PCoA) and permutational multivariate analysis of variance (PERMANOVA) with the ADONIS function based on the weighted UniFrac distance were performed to evaluate the overall differences in the bacterial community (Wang et al. 2018).

## Results

### Effects of microflora source and liquid volume on corn stover degradation

**Fig. 1:**
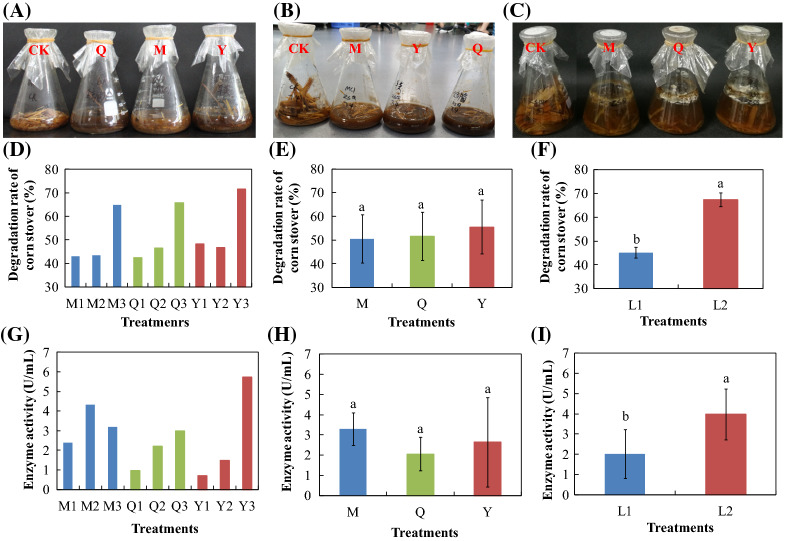
Degradation experiment of the first (**A**), second (**B**), and third (**C**) generation and the effect of treatments (**D**/**G**), microflora sources (**E**/**H**), and liquid volume (**F**/**I**) on corn stover degradation (**D**/**E**/**F**) and endo-glucanase activity (**G**/**H**/**I**). M, microbial consortia source from MC1; Q, microbial consortia source from MC1, sheep, and cattle feces; Y, microbial consortia source from sheep feces; L1, 100 mL PCS medium; L2, 200 mL PCS medium. Values followed by different letters are significantly different at *P* < 0.05

The results showed that the degradation rate of corn stover was significantly affected by liquid volume but not microflora source (Fig. 1D–F). The degradation rate of 200 mL liquid volume (L2) was significantly higher than that of 100 mL (L1) (Fig. 1F). The degradation rate of L2 was increased by 49 % compared with L1, reaching 67.41 % (Fig. 1F). The best degradation treatment was Y3, for which the degradation rate was 71.59 % (Fig. 1D).

### Effects of microflora source and liquid volume on cellulase activities

Endo-glucanase activity was significantly affected by liquid volume but not microflora source (Fig. 1G–I). The endo-glucanase of L2 was significantly increased by 2-fold compared with L1, reaching 3.98 U/ml (Fig. 1I). Pearson correlation results showed that there was a significant correlation between degradation rate and endo-glucanase activity (*P* < 0.05) (Table 2).

**Table 2:** Pearson correlations of degradation ratio with cellulose activity and microbial diversity

		Endoglucanase activity	Bacterial alpha diversity	Fungal alpha diversity
Degradation rata	Pearson correlations	**0.676**	0.136	− 0.422
	Significance (*P*)	**0.046**	0.728	0.258

### Effects of microflora source and liquid volume on microbial diversity

**Fig. 2:**
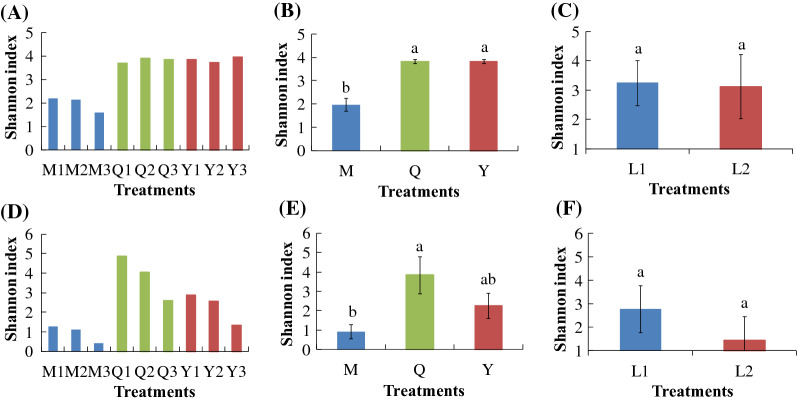
Effect of treatments (**A**/**D**), microflora source (**B**/**E**), and liquid volume (**C**/**F**) on bacterial (**A**/**B**/**C**) and fungal (**D**/**E**/**F**) α-diversity. M, microbial consortia source from MC1; Q, microbial consortia source from MC1, sheep, and cattle feces; Y, microbial consortia source from sheep feces; L1, 100 mL PCS medium; L2, 200 mL PCS medium. Values followed by different letters are significantly different at *P* < 0.05

**Fig. 3:**
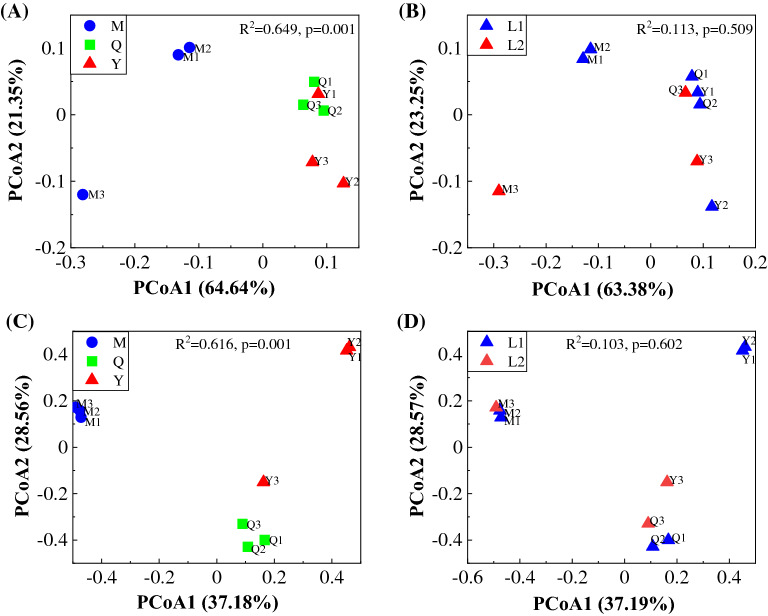
Principal coordinate analysis (PCoA) of weighted UniFrac distances of the bacterial (**A**/**B**) and fungal (**C**/**D**) community under different microflora sources (**A**/**C**) and liquid volumes (**B**/**D**). M, microbial consortia source from MC1; Q, microbial consortia source from MC1, sheep, and cattle feces; Y, microbial consortia source from sheep feces; L1, 100 mL PCS medium; L2, 200 mL PCS medium

Across all samples, we obtained high-quality bacterial (60,162–69,939 sequences per sample, total = 590,998, mean = 65,666) and fungal sequences (53,598–69,121 sequences per sample, total = 564,656, mean = 62,740). After rarefied to 49,000 sequences per sample, microbial diversity and abundance were calculated. The α-diversity of bacteria and fungi was significantly affected by different microflora sources (Fig. 2). The bacterial Shannon index of Q and Y was significantly higher than that of M. The fungal Shannon index of M was significantly lower than that of Q. Liquid volume had no significant effect on microbial α-diversity. Pearson correlation results showed that there was no significant correlation between degradation rate and microbial α-diversity (Table 2). The results of PCoA and PERMAVONA showed that there were significant differences in bacterial and fungal communities of different microflora sources (Fig. 3A and C). The bacterial and fungal communities were not significantly affected by different liquid volumes (Fig. 3B and D).

### Effects of microflora source and liquid volume on microbial composition

**Fig. 4:**
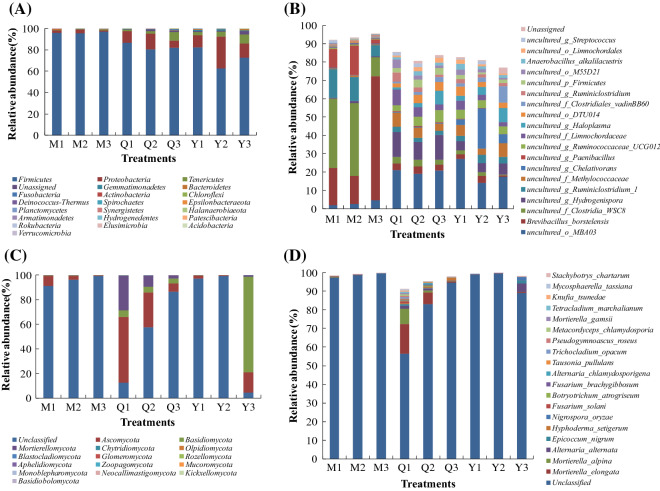
The bacterial (**A**/**B**) and fungal (**C**/**D**) phyla (**A**/**C**) and top 100 species (**B**/**D**) in different treatments

*Firmicutes* and *Proteobacteria* were the dominant bacterial phyla across all treatments (Fig. 4A). The top 5 bacterial species in M were *Brevibacillus borstelensis*, uncultured_*Clostridia*_WSC-8, uncultured_*Ruminiclostridium*_1, uncultured_ *Paenibacillus*, and uncultured_o_*MBA03*. The top 5 bacterial species in Q were uncultured_o_*MBA03*, uncultured_*Hydrogenispora*, uncultured_*Limnochordaceae*, uncultured_*Methylococcaceae*, and uncultured_*Ruminococcaceae*_UCG-012. The top 5 species genera in Y were uncultured_o_*MBA03*, uncultured_*Chelativorans*, uncultured_*Methylococcaceae*, uncultured_*Hydrogenispora*, and uncultured_*Haloplasma* (Fig. 4B). Unclassified, *Ascomycota*, *Basidiomycota*, and *Mortierellomycota* were the dominant fungal phyla across all treatments (Fig. 4C). The top 5 fungal species in M were Unclassified, *Alternaria alternata*, *Fusarium solani*, *Mortierella alpine*, and *Malassezia restricta*. The top 5 fungal species in Q were Unclassified, *Mortierella elongata*, *Mortierella alpina, Alternaria alternata*, and *Hyphoderma setigerum*. The top 5 fungal species in Y were Unclassified, *Alternaria alternata*, *Nigrospora oryzae*, *Epicoccum nigrum*, and *Zopfiella marina* (Fig. 4D).

### Effects of microflora source and liquid volume on microbial taxa

**Fig. 5:**
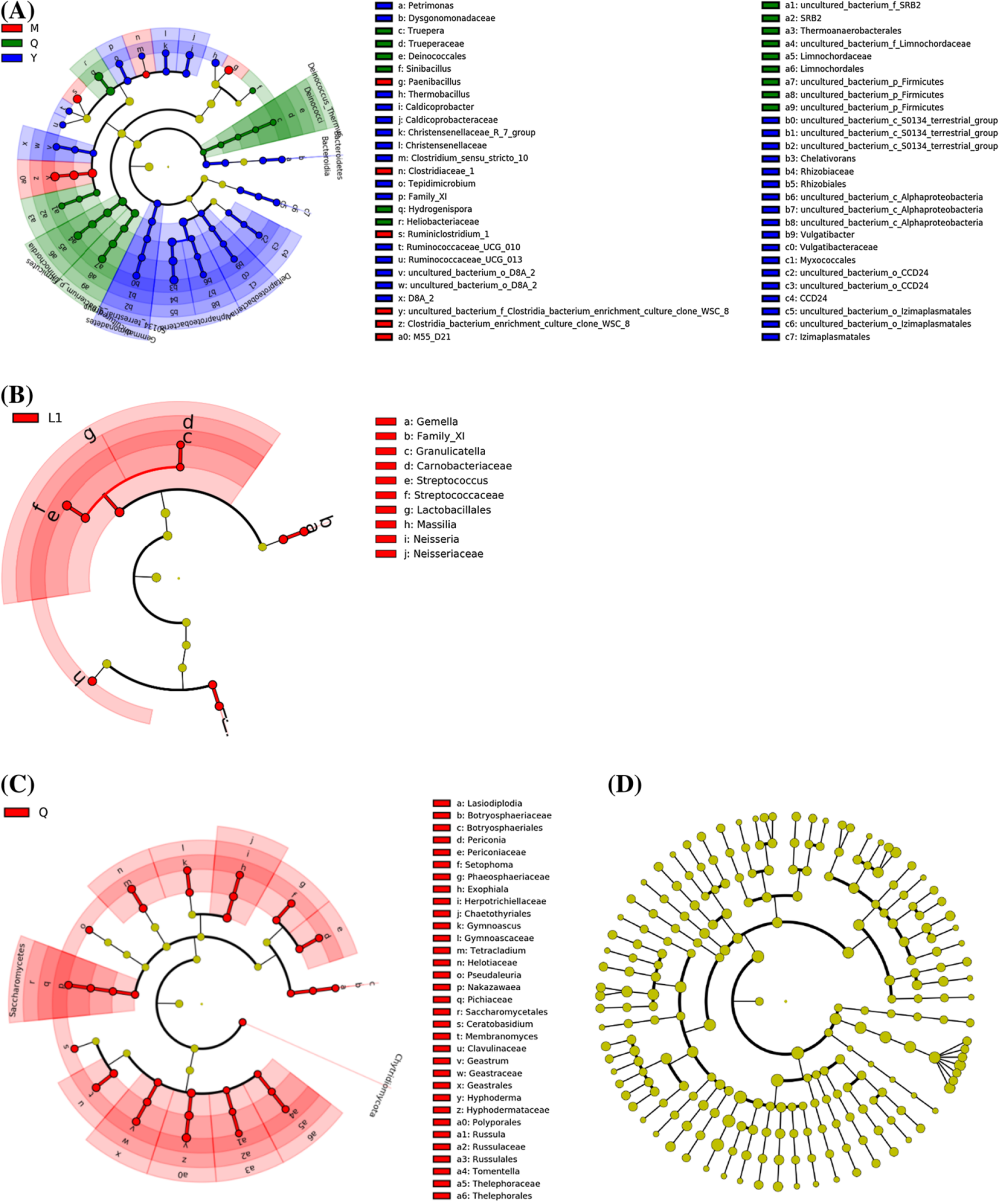
LEfSe analysis of bacterial (**A**, **B**) and fungal (**C**, **D**) composition changes following different microflora sources (**A**/**C**) and liquid volumes (**B**/**D**) (LDA > 2). M, microbial consortia source from MC1; Q, microbial consortia source from MC1, sheep, and cattle feces; Y, microbial consortia source from sheep feces; L1, 100 mL PCS medium; L2, 200 mL PCS medium

LEfSe analysis showed that different microflora sources had significant effects on bacterial taxa (Fig. 5A). M enriched some bacterial taxa from *Paenibacillus*, *Clostridiaceae*_1, *Ruminiclostridium*_1, and *M55_D21*. Q enriched some bacterial taxa from *Deinococcales*, *Sinibacillus*, *Heliobacteriaceae*, *Thermoanaerobacterales*, *Limnochordales*, and uncultured_bacterium_p_*Firmicutes*. Y enriched some bacterial taxa from *Dysgonomonadaceae*, *Thermobacillus*, *Caldicoprobacteraceae*, *Christensenellaceae*, *Clostridium_sensu_stricto_10*, Family_*XI*, *Ruminococcaceae_UCG_010*, *Ruminococcaceae_UCG_013*, *D8A_2*, uncultured_*S0134*, *Rhizobiales*, uncultured_*Alphaproteobacteria*, *Myxococcales*, *CCD24*, and *Izimaplasmatales*. There were a few differences in bacterial taxa between different liquid volumes (Fig. 5B). L1 enriched 15 bacterial species from *Gemella*, XI, *Granulicateriaceae*, *Streptococcaceae*, *Lactobacillales*, *Massilia*, and *Neisseriaceae*. Different microflora sources had an effect on fungal taxa (Fig. 5C). Q enriched 44 fungal taxa from *Botryosphaeriales*, *Periconiaceae*, *Phaeosphaeriaceae*, *Alternaria chlamydosporigena*, *Chaetothyriales*, *Gymnoascaceae*, *Helotiaceae*, *Pseudaleuria*, *Saccharomycetes*, *Fusarium brachygibbosum*, *Chaetomium iranianum*, *Microdochium trichocladiopsis*, *Ceratobasidium*, *Clavulinaceae*, *Geastrales*, *Polyporales*, *Russulales*, *Thelephorales*, *Chytridiomycota*, *Mortierella amoeboidea*, and *Mortierella hyalina*. There was no significant difference in fungal taxa between different liquid volumes (Fig. 5D).

## Discussion

Our study revealed that microflora source had no significant effect on corn stover degradation. However, Wongwilaiwalin et al. (2013) showed that microbial consortia enriched from industrial sugarcane bagasse pile (BGC-1) had a better ability to degrade alkali pretreated corn stover (72 %) than those enriched from cow rumen fluid (CRC-1) and pulp mill-activated sludge (ASC-1). Different conclusions on degradation rate might be due to the similarity of consortia sources. All three of our microbial consortia (M/Q/Y) came from feces or compost containing feces. The sources of our microbial consortia is similar, while the sources of Wongwilaiwalin’s microbial consortia is different. This indicated that the ability of lignocellulose degradation rate might not be very different among the microbial consortia from similar sources.

Our study found that a higher liquid volume increased corn stover degradation. Higher liquid volume will lead to lower dissolved oxygen in the conical flask. The initial surface dissolved oxygen of L1 and L2 was 7.05 and 5.86 mg/L, respectively. This indicated that straw degradation was more efficient in a lower dissolved oxygen environment. This was supported by previous works demonstrating that microbial consortia need a microaerobic environment to degrade corn stover (Lu et al. 2008; Wang et al. 2004). Some reports also displayed that straw degradation was completed by aerobic and anaerobic bacteria, in which anaerobic bacteria played a role in degradation (Kato et al. 2005; Zhou et al. 2015). Therefore, we speculated that the lower dissolved oxygen environment caused by a higher liquid volume might promote the abundance or activity of anaerobic lignocellulose-degrading bacteria.

There are many reports on microbial consortia degrading corn stover (Table 1). The best microbial consortia can degrade 62 % of corn stover without pretreatment and 78 % of corn stover pretreated with acid or alkali (Wongwilaiwalin et al. 2010; Zhang et al. 2018). Our results showed that the three microbial consortia (M/Q/Y) degraded more than 60 % of corn stover without pretreatment, and the highest degradation rate was 71.59 % by Y3. This indicated that the microbial consortia from feces could degrade corn straw efficiently, and the microbial consortia from sheep feces were slightly better than others.

Microorganisms hydrolyze straw to monomeric sugars by cellulase. Cellulase is a multienzyme complex mainly including endo-glucanase, exo-glucanase, and β-glucosidase, which act synergistically during enzymatic hydrolysis (Saini et al. 2015). Endo-glucanase is the most important component of cellulase system (Zhang et al. 2019). Endo-glucanase (EC 3.2.1.4) can randomly cleave the internal beta-1,4-glycosidic bonds in amorphous regions of cellulose polymers. In this study, endo-glucanase was used as an important basis to judge the ability of cellulase. The results showed that liquid volume had a significant effect on endo-glucanase activity, and endo-glucanase was closely related to degradation rate, which is supported by previous works (Takizawa et al. 2020; Wang et al. 2004). This indicated that the lower dissolved oxygen environment caused by a higher liquid volume promote the activity of anaerobic lignocellulose degrading bacteria.

There are few studies on the effect of liquid volume on microbial diversity. Our study showed that the liquid volume had a significant effect on the degradation rate but not on microbial diversity. Microbial diversity was not closely related to the degradation rate. This suggested that the lower dissolved oxygen environment caused by higher liquid volume mainly increased the degradation rate of corn straw by promoting microbial activity but not microbial diversity. Our study also revealed that the microflora source had a significant effect on microbial diversity but not degradation rate. This indicated that the species degrading corn straw may be diverse. However, a study revealed that the microflora source had a significant effect on degradation rate but not on microbial diversity (Wongwilaiwalin et al. 2013). Different conclusions on degradation rate might be due to the similarity of consortia sources. We have already discussed this point in the first paragraph of Discussion. Different conclusions on microbial diversity might be due to different domestication times. The domestication time of our microbial consortia was short (4–6 generations), while that of Wongwilaiwalin’s was long (21–27 generations). With the extension of domestication time and convergence adaptation, the microbial diversity might be more similar. This also indicated that different spatiotemporal scales might lead to different conclusions. Wongwilaiwalin’s conclusion might be of larger spatiotemporal scale, and ours might be of smaller spatiotemporal scale.

A few studies analyzed the bacterial composition of corn stover degrading microbial consortia by high-throughput sequencing technology. In this study, we showed that the three microbial consortia (M, Q, and Y) of corn straw degradation were mainly composed of *Firmicutes* and *Proteobacteria*. The results were supported by some previous works (Feng et al. 2011; Hua et al. 2014; Yu et al. 2019). However, some reports also showed that the microbial consortia degrading corn stover were mainly composed of *Proteobacteria* and *Bacteroidetes* or *Firmicutes* and *Bacteroidetes* or *Proteobacteria* and *Actinobacteria* (Liu et al. 2010; Qiao et al. 2013). These differences may be caused by the different sources and culture conditions of the microbial consortia. The microbial consortia composed of *Firmicutes* and *Proteobacteria* mainly came from feces and compost, and that composed of other bacteria mainly came from soil. Bacterial composition at the species level showed that most of the species in M, Q, and Y were unculturable bacteria. The dominant species in M, Q, and Y were significantly different. Uncultured_o_*MBA03* was a common dominant species in M, Q, and Y. MBA03 is often found in thermophilic anaerobic environments and may have a strong ability to degrade lignocellulose (Wu et al. 2020). *Brevibacillus borstelensis*, uncultured_*Clostridia*_*WSC-8*, uncultured_*Ruminiclostridium*_1, and uncultured_ *Paenibacillus* in M and uncultured_*Ruminococcaceae_UCG-012* in Q have been reported to degrade lignocellulose (Liang et al. 2009; Mathews et al. 2016; Zhang et al. 2012).

Few studies have analyzed the fungal composition of corn straw degrading microbial consortia using high-throughput sequencing technology. This study showed that dominant fungi in M, Q, and Y were unclassified, *Ascomycota*, *Zygomycota*, and *Mortierellomycota*. *Ascomycota* and *Zygomycota* were also reported as dominant fungi in corn stover degrading microbial consortia CCS-1 by a clone library (Liu et al. 2010). Fungal composition at the species level showed that most of the species in M, Q, and Y were unclassified, and the dominant species in M, Q, and Y were significantly different. *Alternaria alternata*, which is a common dominant species in M, Q, and Y, has the ability to degrade cellulose and lignin (Guillen et al. 1987; Sharma et al. 2016). *Fusarium solani* in M, *Mortierella elongata* and *Hyphoderma setigerum* in Q, *Nigrospora oryzae* and *Epicoccum nigrum* in Y have been reported to have the ability to degrade lignocellulose (Lozovaya et al. 2006; Olajuyigbe et al. 2016; Yao et al. 2012; Yurchenko and Wu 2014).

The species enriched or inhibited by different microflora sources have close phylogenetic relationships, such as *Paenibacillus* enriched in M, *Sinibacillus* enriched in Q, and *Thermobacillus* enriched in Y that all belong to *Bacillales*. M enriched *Clostridiaceae*_1, *Ruminiclostridium_1*, and *M55_D21*, Q enriched *Heliobacteriaceae* and *Thermoanaerobacterles*, and Y enriched *Caldicoprobacteraceae*, *Christensenellaceae*, *Clostridium_sensu_stricto_10*, Family_*XI*, *Ruminococcceae_UCG_010*, *Ruminococcceae_UCG_013*, and *D8A_2*, which all belong to *Clostridia*. It is speculated that the functions of these species in the three bacterial communities may be similar. Most of these species in *Bacillales* (Kong et al. 2020; Mathews et al. 2016) and *Clostridia* (Fosses et al. 2017; Meng et al. 2020) have stress resistance, such as high temperature and low dissolved oxygen resistance, and the potential for lignocellulose degradation. This was also supported by previous works that consortia originated from highly diverse environmental microflora sharing similar composite profiles at higher taxa levels with substantial differences at lower taxa levels (Wongwilaiwalin et al. 2013). In addition, Q and Y also enriched some thermophilic, anaerobic, or cellulolytic bacteria (Garcia and Müller 2020; Puig-Castellvi et al. 2020). Bacterial species enriched by L1 were mainly (facultative) aerobic bacteria (Van Craenenbroeck et al. 2011; Zotta et al. 2017), and most of these species cannot degrade cellulose. This indicated that the aerobic bacteria decreased significantly with the increase of liquid volume. The fungi enriched by Q were mainly from *Ascomycota* and *Basidiomycota*. Among them, some species in *Polyporales* and *Helotiaceae* have the ability to degrade lignocellulose (Gianoulis et al. 2012; Huang et al. 2019).

**Fig. 6:**
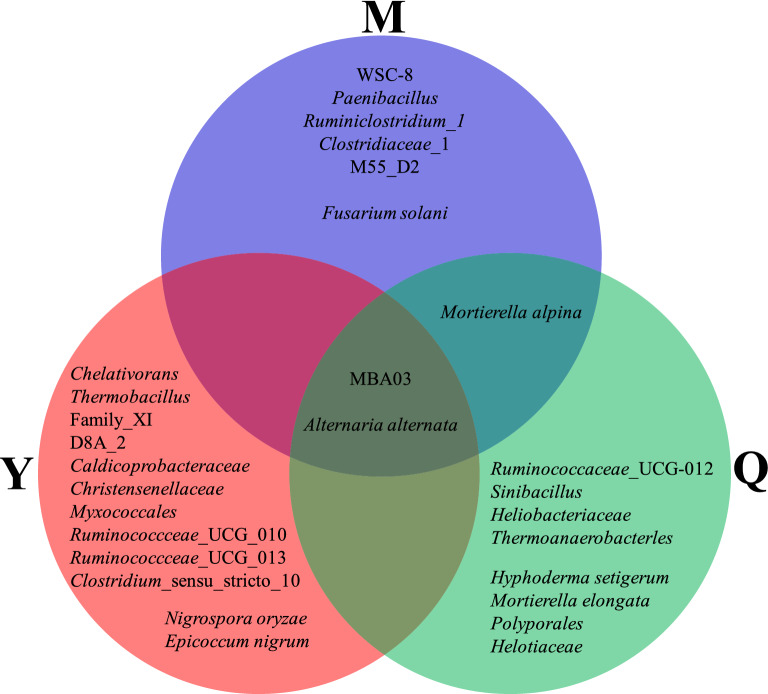
Dominant and enriched species with a lignocellulosic degradation ability among the three bacterial communities. M, microbial consortia source from MC1; Q, microbial consortia source from MC1, sheep, and cattle feces; Y, microbial consortia source from sheep feces

The dominant and enriched species with the lignocellulosic degradation ability among the three bacterial communities (M/Q/Y) were sorted out (Fig. 6). It suggested that different microbial communities might degrade corn straw through different species combinations. Figure 3A showed that the distance between Q and Y bacterial community was close, and that between Q and M bacterial community was far. It was speculated that most of the bacteria in Q might originate from Y. Figure 3C showed that the fungal communities of Q and Y3 are close, and Q was far away from other samples. It was speculated that most of the fungi in Q might originate from N (it was a pity that N samples with weak degradation ability were not reserved for microbial diversity detection), and some of them might originate from Y. However, Fig. 6 showed that Q, M and Y shared less enriched species. It was speculated that when M, Y and N were mixed into Q, the complex interaction between species made their abundance changed significantly, resulting in that although the bacterial communities of Q and Y were similar, the enriched species were obviously different. The relationship between mixed microflora (e.g. Q) and source microflora (e.g. M, Y and N) is worthy of further study.

In conclusion, our results show that liquid volume had a significant effect on degradation rate while microflora source had a significant effect on microbial community in corn stover degradation.

## Data Availability

The raw sequence data reported in this paper have been deposited in the Genome Sequence Archive (Wang et al. 2017) in National Genomics Data Center (Zhang et al. 2020), Beijing Institute of Genomics (China National Center for Bioinformation), Chinese Academy of Sciences, under accession number CRA003666 that are publicly accessible at https://bigd.big.ac.cn/gsa.
